# Visit-to-visit variability of glycated albumin was associated with incidence or progression of lower extremity atherosclerotic disease

**DOI:** 10.1186/s12933-020-01187-1

**Published:** 2020-12-10

**Authors:** Yun Shen, Dongjun Dai, Jingyi Lu, Yufei Wang, Wei Zhu, Yuqian Bao, Gang Hu, Jian Zhou

**Affiliations:** 1 Department of Endocrinology and Metabolism, Shanghai Jiao Tong University Affiliated Sixth People’s Hospital, Shanghai Clinical Center for Diabetes, Shanghai Diabetes Institute, Shanghai Key Laboratory of Diabetes Mellitus, 600 Yishan Road, Shanghai, 200233 China; 2 Chronic Disease Epidemiology Laboratory, Pennington Biomedical Research Center, 6400 Perkins Road, Baton Rouge, LA 70808 USA

**Keywords:** Glycated albumin, Glycemic variability, Lower extremity atherosclerotic disease

## Abstract

**Background:**

The aim of this study was to investigate the association of visit-to-visit variability of hemoglobin A1c (HbA1c) and glycated albumin (GA) with the risk of lower extremity atherosclerotic disease (LEAD).

**Method:**

We performed a prospective cohort study of 436 patients with type 2 diabetes (258 men and 178 women) with at least 3 measurements of HbA1c and GA prior to baseline investigation from the Department of Endocrinology and Metabolism, Shanghai Sixth People’s Hospital. Different HbA1c and GA variability markers were calculated. Multivariable Cox proportional hazard regression models were used to demonstrate the association between visit-to-visit HbA1c and GA variability and the risk of incident or progressive LEAD.

**Results:**

During a mean follow-up period of 3.77 years, 112 participants developed LEAD. Multivariate-adjusted hazard ratios (HRs) of LEAD across tertiles of GA-CV values were 1.00, 1.06 (95% confidence interval [CI] 0.65–1.75), and 1.71 (95% CI 1.07–2.73) (*P* for trend = 0.042), respectively. When we used GA-VIM and GA-ARV values as exposures, similar positive associations with the risk of LEAD primary were found. Multivariate-adjusted HRs of LEAD for each 1 unit increase in GA-CV, GA-VIM and GA-ARV were 1.03 (95% CI 1.01–1.06), 1.32 (95% CI 1.03–1.69), and 1.07 (95%CI 1.01–1.15), respectively. However, there was no significant association between visit-to-visit variability of HbA1c and the risk of LEAD.

**Conclusions:**

Visit-to-visit variability of GA may be an optimal biomarker in relation to LEAD risk among patients with type 2 diabetes.

## Background

Lower extremity atherosclerotic disease (LEAD) is one of the life-threatening complications of diabetes. LEAD and the subsequent vascular occlusion, gangrene and lower extremity amputation seriously affect the quality of life of patients and increase the economic burden [1, 2]. However, the onset of LEAD is insidious, and 40% of patients are asymptomatic. Therefore, early identification and intervention to delay the progression of LEAD can effectively reduce the risk of the above serious outcomes.

Among patients with diabetes, long-term exposure to hyperglycemia is an important risk factor for the development of macrovascular complications. Glycated hemoglobin (HbA1c) has been widely used as a marker for glycemic control [3, 4]. Previously, we have demonstrated the association between visit-to-visit HbA1c variability and the incidence of both coronary heart disease and stroke by real world data. Severe hypoglycemia events may partly mediate the above association [5]. However, based on recent findings, HbA1c is insufficient in terms of the overall evaluation of glycemic control [6]. Recently, glycated albumin (GA) is also considered as an indicator that reflects long-term glycemic control. Compared with HbA1c, GA is not affected by the measurement of hemoglobin, and studies have found that GA may more accurately reflect long-term glycemic fluctuations in postprandial glucose levels than HbA1c [7], while most patients with diabetes in China are featured with postprandial hyperglycemia. In addition, cohort studies with long term follow up regarding LEAD as the primary outcome are limited.

Given the above reason, we speculate that visit-to-visit variability of GA can provide more information on long-term glycemic control than HbA1c. The association between visit-to-visit variability of GA and LEAD in patients with type 2 diabetes remains uncertain. The present study aimed to investigate the association between visit-to-visit variability of GA and the risk of LEAD in patients with type 2 diabetes.

## Methods

### Study design, sample size calculation and participants

This is a prospective and dynamic cohort study of patients with type 2 diabetes aged 18–80 years who were admitted to the Department of Endocrinology and Metabolism, Shanghai Sixth People’s Hospital from January 2011 to September 2019. All patients were previously confirmed with type 2 diabetes by the 1999 World Health Organization criteria [8]. Eligible patients should have at least 3 measurements of GA and HbA1c within 2 years before baseline data collection. We calculated the sample size prior to the recruitment at baseline based on the findings from a pilot study. A Cox regression of the log hazard ratio on a covariate with a standard deviation of 7.3 of GA-CV based on a sample of 400 observations achieves 80% power at a 0.05 significance level to detect a regression coefficient equal to 0.04. The sample size was adjusted for an anticipated event rate of 0.26. Considering a 20% of loss during follow-ups, a total of 611 eligible patients with type 2 diabetes were screened at baseline to ensure a sufficient sample size. The average duration of diabetes was 11.5 ± 6.49 years. We excluded patients with a history of malignancy or mental disorders (n = 53), end-stage renal disease (n = 1), onset of diabetic ketoacidosis (n = 6), a history of congestive heart failure and lower extremity amputation at baseline (n = 12). Finally, a total of 539 patients were enrolled in the study at baseline (see Additional file 1: Figure S1). The study and the analysis plan were approved by the Institutional Review Boards (Research Ethics Committees) of Shanghai Sixth People’s Hospital. We have obtained informed consent from all participants.

### Baseline anthropometric and laboratory measurements

All participants underwent routine physical examination to measure height, body weight, waist circumference and blood pressure. Body mass index (BMI) was calculated as body weight in kilograms divided by square of height in meters. Waist circumference was measured by a tape around the horizontal plane between the inferior costal margin and the iliac crest on the mid-axillary line with the subject in a standing position. Blood pressure was measured twice with a mercury sphygmomanometer, and the average value of the two measurements was taken. History of comorbidities and prescriptions was collected including anti-hypertensive medications, glucose-lowering medications, lipid-lowering medications and antiplatelet medications. Using data collected on the patient’s self-reported smoking status, we classified the patients into 2 groups: current smokers, non-current smokers including ever smokers and never smokers. Current alcohol consumption was defined as weekly alcohol consumption over 140 g in men and 70 g in women [9]. Mean value of HbA1c was defined as the one before the baseline data collection.

All subjects were fasted overnight for 8–10 h before venous blood sampling. Serum uric acid (UA), serum creatinine (Scr), total cholesterol (TC), triglycerides (TG), low-density lipoprotein cholesterol (LDL-C), high density lipoprotein cholesterol (HDL-C), fasting blood glucose (FPG) and fasting C peptide (FCP) were measured using the same methods that have been previously described [10]. GA was measured via an enzymic method (Lucica GA-L, Asahi Kasei Pharma, Tokyo, Japan) with the 7600-120 auto-analyzer (Hitachi, Tokyo, Japan). The inter- and intra- batch coefficients of variation were 5.1% and 3.0% respectively. HbA1c was measured via high performance liquid chromatography (Variant II, Bio-Rad, Hercules, CA, USA). The inter- and intra- batch coefficients of variation were 3.4% and 2.6% respectively. The estimated glomerular filtration rate (eGFR) was estimated using the Chronic Kidney Disease Epidemiology Collaboration equation (CKD-EPI) [11].

### Visit-to-visit variability of GA and HbA1c

The updated mean value, standard deviation (SD), variability independent of the mean (VIM), and the average real variability (ARV) of GA and HbA1c were calculated for each participant within 2 years before baseline date collection. CV of GA and HbA1c was calculated as SD/mean × 100%. VIM was calculated as SD/mean^β^. β stands for a standardized coefficient of fitted power function with SD and mean. ARV was calculated as the average value of the difference between consecutive measurements [12]. In this study, the average numbers of measurements of GA and HbA1c during an average of 1.4 years before baseline were 3.6 and 3.8, respectively.

### Prospective follow-up

The primary outcome (LEAD) was the composite outcome of either incident LEAD or progressive LEAD confirmed by Doppler ultrasonography. The femoral artery, deep femoral artery, popliteal artery, superficial femoral artery, anterior tibial artery, posterior tibial artery and peroneal artery were measured bilaterally. Arterial intima-media thickness (IMT) was measured as the distance between the intima-lumen and the media-adventitia [13]. Atherosclerotic plaque was defined as a focal lesion that invaded the arterial lumen at least 0.5 mm, or exceeded the surrounding IMT value by 50%, or IMT ≥ 1.5 mm [14]. Patients with LEAD were confirmed by atherosclerotic plaque in any of the above-mentioned lower limb arterial segments [15]. Based on the above methodology, incident LEAD was defined as the development of new-onset atherosclerotic plaque in patients without LEAD at baseline. Progressive LEAD was defined as the expansion of lower extremity atherosclerotic plaque or the development of new lower extremity atherosclerotic occlusion in patients with LEAD at baseline. The secondary outcomes included incident LEAD and progressive LEAD individually. The duration of follow-up for each patient was calculated from the baseline date to the date of diagnosis of the outcome, the date of dropout, the date of death, or June 30, 2020.

### Statistical analyses

The associations between visit-to-visit variability of GA and HbA1c (CV, VIM and ARV) and the risk of the primary outcome were analyzed by use of Cox proportional hazards models. CV, VIM and ARV of GA and CV of HbA1c were evaluated in the following 2 ways: as tertiles and as a continuous variable (per 1-unit increase). These visit-to-visit GA and HbA1c variability metrics were included in the models as dummy variables, and the significance of the trend across categories of GA and HbA1c was tested in the same models by giving an ordinal numeric value for each dummy variable. The proportional hazards assumption in the Cox model was assessed with graphical methods and with models including time-by-covariate interactions. In general, all proportionality assumptions were appropriate. All analyses were conducted after adjusting for age and sex, and then a backward stepwise selection procedure was used for candidate covariates in the multivariable model including BMI, waist circumference, diabetes duration, systolic blood pressure (SBP), lipid profile (TG, LDL-C and HDL-C), eGFR, smoking status, current alcohol drinking, insulin therapy, anti-hypertensive therapy, aspirin, statins therapy, and mean value of HbA1c. Finally, we included diabetes duration, smoking status, eGFR, HDL-C, mean value of HbA1c and use of aspirin in the multivariable-adjusted model. Statistical significance was considered to be *P* < 0.05. All statistical analyses were performed using IBM SPSS Statistics for Windows, version 24.0 (IBM Corp., Armonk, NY, USA).

## Results

A total of 539 patients were enrolled in the study at baseline with a loss of 103 during follow-ups. Finally, 436 patients with type 2 diabetes were included into the final analysis. Compared with patients with diabetes excluded in the present study, the patients with type 2 diabetes included in the present study had the similar age (58.6 versus 59.9 years old, *P* = 0.324), and fewer men (59.2% versus 67.0%, *P* < 0.05). The baseline characteristics are listed in Table 1 based on the presence of LEAD at baseline. Patients with LEAD at baseline were older, with longer duration of diabetes, and had higher systolic BP, worse renal function, lower total cholesterol, and were more likely to receive insulin, aspirin and statin therapies compared with patients without LEAD at baseline.

**Table 1:** Baseline characteristics of study participants

Variables	All subjects	With LEAD at baseline	Without LEAD at baseline	*P* value
No. of subjects	436	194	242	–
Male, %	59.2	64.9	52.1	0.007
Age, years	58.6 ± 10.3	63.4 ± 7.92	52.6 ± 9.65	< 0.001
Diabetes duration, years	11.7 ± 6.51	13.4 ± 6.55	9.63 ± 5.84	< 0.001
Body mass index, kg/m^2^	25.4 ± 2.98	25.3 ± 3.08	25.5 ± 2.86	0.583
Waist, cm	92.0 ± 8.92	92.5 ± 8.68	91.3 ± 9.20	0.154
Systolic blood pressure, mmHg	133 ± 16.1	135 ± 16.7	128 ± 14.8	< 0.001
Diastolic blood pressure, mmHg	79.2 ± 8.65	79.2 ± 8.51	79.3 ± 8.85	0.867
HbA1c, mmol/mol	63.8 ± 17.1	64.7 ± 16.2	62.6 ± 18.1	0.188
HbA1c, %	7.99 ± 1.57	8.08 ± 1.49	7.88 ± 1.66	0.188
GA, %	19.8 ± 5.81	20.0 ± 5.58	19.6 ± 6.04	0.566
Fasting Plasma glucose, mmol/L	7.62 ± 2.41	7.48 ± 2.27	7.80 ± 2.56	0.174
Fasting C-peptide, ng/mL	2.10 ± 1.53	2.02 ± 1.06	2.19 ± 1.96	0.248
Current smoker, %	27.5	29.3	25.3	0.343
Alcohol drinker, %	13.1	14.0	11.9	0.499
eGFR, mL/(min 1.73m^2^)	93.6 ± 17.9	87.7 ± 17.6	101 ± 15.2	< 0.001
Uric acid, μmol/L	335 ± 83.1	341 ± 83.7	330 ± 80.8	0.169
Total cholesterol, mmol/L	4.48 ± 1.15	4.35 ± 1.30	4.64 ± 0.90	0.007
Triglyceride, mmol/L	1.85 ± 2.36	1.79 ± 2.82	1.93 ± 1.61	0.534
LDL cholesterol, mmol/L	2.61 ± 0.88	2.54 ± 0.95	2.70 ± 0.79	0.051
HDL cholesterol, mmol/L	1.08 ± 0.30	1.06 ± 0.27	1.11 ± 0.33	0.093
Use antidiabetes agents, %				
Biguanides	49.5	43.0	57.7	0.002
Sulfonylureas	33.1	31.0	37.6	0.146
Thiazolidinediones	8.94	7.85	10.3	0.371
Glinides	13.1	10.3	16.5	0.058
DPP-4 inhibitors	4.59	4.55	4.64	0.963
Glucosidase inhibitors	62.8	68.6	55.7	0.006
SGLT-2 inhibitors	0.00	0.00	0.00	-
GLP-1 receptor agonists	1.15	0.41	2.06	0.108
Insulin, %	61.7	66.1	56.2	0.034
Use antihypertension agents, %				
RAAS inhibitors	48.9	55.4	40.7	0.002
Calcium-channel blockers	30.7	36.0	24.2	0.008
β-Blockers	18.8	22.7	13.9	0.019
Diuretics	6.65	10.7	1.55	< 0.001
Use lipid-lowering agents, %				
Statins	56.2	66.9	42.8	< 0.001
Fibrates	7.57	6.20	9.28	0.237
Aspirin, %	49.8	60.7	36.1	< 0.001
Anticoagulant, %	0.00	0.00	0.00	–
Mean HbA1c, mmol/mol	62.9 ± 15.1	64.4 ± 14.9	61.1 ± 15.1	0.025
Mean HbA1c, %	7.91 ± 1.38	8.05 ± 1.36	7.75 ± 1.38	0.025
Mean GA, %	19.7 ± 4.70	20.0 ± 4.66	19.3 ± 4.72	0.101
Variability of GA				
CV	11.1 ± 7.20	11.9 ± 6.90	10.2 ± 7.48	0.017
VIM	1.03 ± 0.74	1.11 ± 0.72	0.94 ± 0.75	0.022
ARV	3.09 ± 2.45	3.27 ± 2.40	2.86 ± 2.48	0.085
Variability of HbA1c				
CV	7.44 ± 5.59	7.53 ± 5.54	7.34 ± 5.66	0.723
VIM	0.23 ± 0.18	0.23 ± 0.18	0.22 ± 0.18	0.503
ARV	0.80 ± 0.72	0.81 ± 0.69	0.79 ± 0.76	0.783

**Fig. 1:**
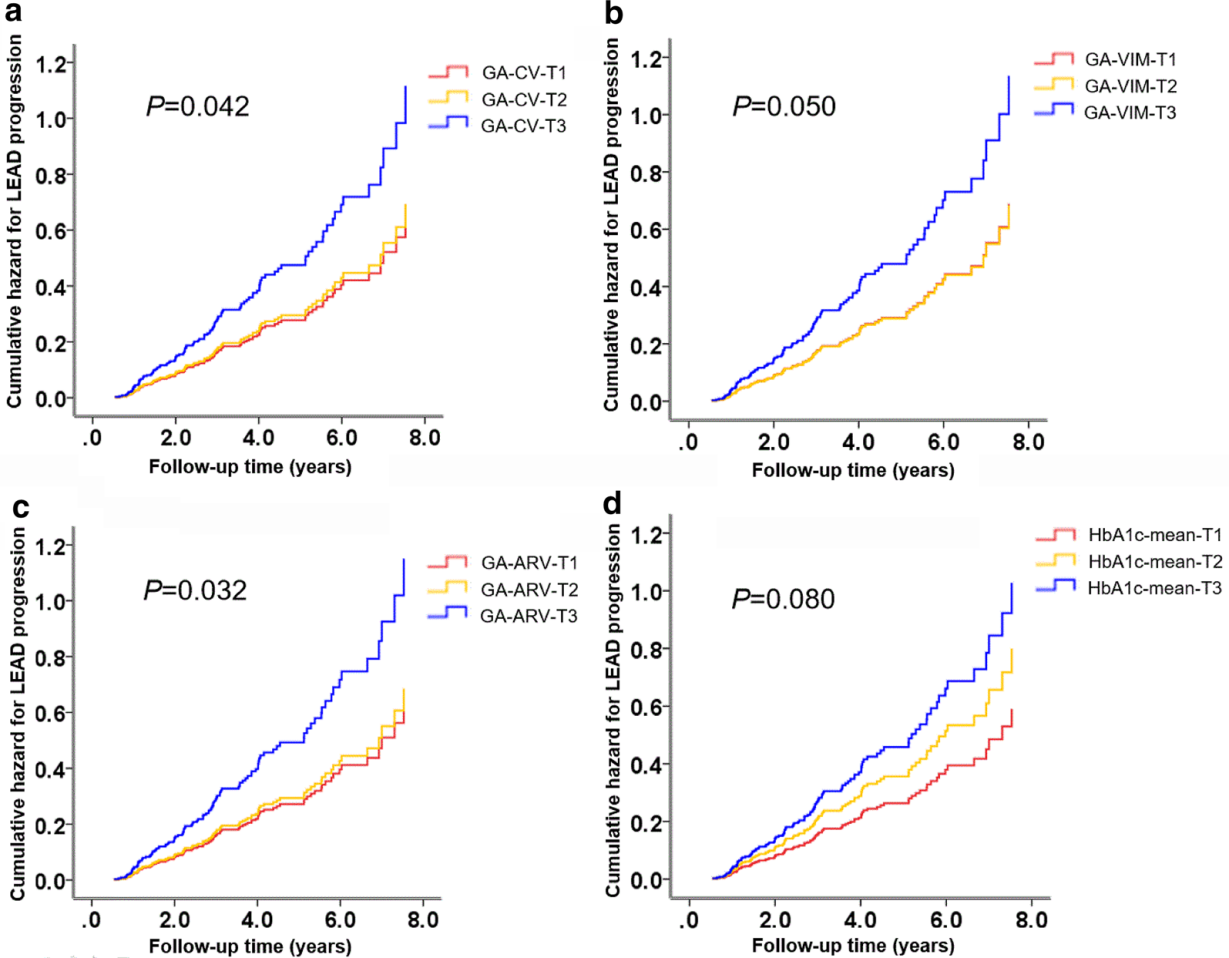
Cumulative incidences of the LEAD progression according to the GA variability indicators (1A-CV, 1B-VIM, 1C-ARV) and mean A1c (1D) tertiles (T1–T3) using the cox proportional hazards regression analysis

During a mean follow-up period of 3.77 years, 112 participants developed the primary outcome. Multivariate-adjusted (diabetes duration, smoking status, eGFR, HDL, aspirin, and mean value of HbA1c) HRs of the primary outcome across tertiles of GA-CV values were 1.00, 1.06 (95% confidence interval [CI] 0.65–1.75), and 1.71 (95% CI 1.07–2.73) (*P* for trend = 0.042), respectively (Table 2). When we used GA-VIM and GA-ARV values as exposures, similar positive associations with the risk of LEAD were found (Table 2). Multivariate-adjusted HRs of LEAD for each 1 unit increase in GA-CV, GA-VIM and GA-ARV were 1.03 (95% CI 1.01–1.06), 1.32 (95% CI 1.03–1.69), and 1.07 (95%CI 1.01–1.15), respectively (Table 2). Figure 1a presented the significant trend of hazard ratios for the primary outcome across tertiles of GA-CV.

**Table 2:** Hazard ratios for the primary outcome based on different GA variability metrics

	GA variability indicators tertiles			*P* value for trend	Per 1 unit increase
	T1	T2	T3		
CV	< 7.35	7.35–12.47	≥ 12.48	–	–
No. of participants	144	145	147	–	–
No. of cases	31	32	49	–	–
Person-years	510	507	475	–	–
Age and sex-adjusted HRs	1.00	1.02 (0.62–1.68)	1.74 (1.10–2.73)	0.019	1.03 (1.01–1.06)
Multivariable adjusted HRs	1.00	1.06 (0.65–1.75)	1.71(1.07–2.73)	0.042	1.03 (1.01–1.06)
VIM	< 0.61	0.61–1.12	≥ 1.13	–	–
No. of participants	146	144	146	–	–
No. of cases	32	31	49	–	–
Person-years	521	521	451	–	–
Age and sex-adjusted HRs	1.00	0.95 (0.58–1.57)	1.85 (1.19–2.89)	0.004	1.40 (1.12–1.76)
Multivariable adjusted HRs	1.00	0.99 (0.60–1.64)	1.65 (1.02–2.67)	0.050	1.32 (1.03–1.69)
ARV	< 1.75	1.75–3.29	≥ 3.30	–	–
No. of participants	145	146	145	–	–
No. of cases	29	33	50	–	–
Person-years	510	523	460	–	–
Age and sex-adjusted HRs	1.00	1.11 (0.67–1.82)	1.97 (1.24–3.12)	0.005	1.10 (1.03–1.18)
Multivariable adjusted HRs	1.00	1.08 (0.64–1.81)	1.81 (1.08–3.04)	0.032	1.07 (1.01–1.15)

Multivariable adjustments included diabetes duration, smoking status, eGFR, HDL, aspirin, and mean HbA1c

Hazard ratios of the primary outcome at different time points are listed in Table 3. The multivariable-adjusted hazard ratios of LEAD by GA-CV within different follow-up years were 0.99 (95% CI 0.91–1.07) within 1 year, 1.02 (95% CI 0.98–1.06) within 2 years, 1.02 (95%CI 0.99–1.05) within 3 years, 1.03 (95% CI 1.01–1.06) within in 4 years, 1.03 (95% CI 1.01–1.05) within 5 years, 1.03 (1.01–1.05) within 6 years, and 1.03 (95% CI 1.01–1.06) for 6 years and over, respectively. However, both GA-VIM and GA-ARV were not reflective of the primary outcome in assessing the risks of the primary outcome at different time points (Table 3).

**Table 3:** Hazard ratios for the primary outcome at different time intervals

	GA-CV	GA-VIM	GA-ARV
No. of participants	436	436	436
< 1 year			
No. of cases	13	13	13
Person-years	298	298	298
Age-adjusted HRs	0.97 (0.90–1.05)	0.85 (0.40–1.78)	1.01 (0.83–1.24)
Multivariable adjusted HRs	0.99 (0.91–1.07)	0.97 (0.44–2.12)	1.05 (0.85–1.30)
< 2 years			
No. of cases	44	44	44
Person-years	543	543	543
Age-adjusted HRs	1.02 (0.98–1.06)	1.21 (0.84–1.74)	1.09 (0.98–1.20)
Multivariable adjusted HRs	1.02 (0.98–1.06)	1.18 (0.79–1.75)	1.08 (0.97–1.21)
< 3 years			
No. of cases	73	73	73
Person-years	725	725	725
Age-adjusted HRs	1.03 (0.99–1.06)	1.33 (1.01–1.77)	1.10 (1.01–1.19)
Multivariable adjusted HRs	1.02 (0.99–1.05)	1.22 (0.90–1.66)	1.06 (0.97–1.16)
< 4 years			
No. of cases	88	88	88
Person-years	852	852	852
Age-adjusted HRs	1.03 (1.01–1.06)	1.33 (1.05–1.75)	1.10 (1.03–1.18)
Multivariable adjusted HRs	1.03 (1.01–1.06)	1.30 (0.99–1.70)	1.07 (0.99–1.15)
< 5 years			
No. of cases	95	95	95
Person-years	929	929	929
Age-adjusted HRs	1.03 (1.01–1.05)	1.36 (1.07–1.73)	1.10 (1.03–1.18)
Multivariable adjusted HRs	1.03 (1.01–1.05)	1.27(0.98–1.64)	1.07 (0.99–1.15)
< 6 years			
No. of cases	106	106	106
Person-years	976	976	976
Age-adjusted HRs	1.03 (1.01–1.06)	1.39 (1.11–1.75)	1.10 (1.03–1.18)
Multivariable adjusted HRs	1.03 (1.01–1.05)	1.29 (1.01–1.66)	1.06 (0.99–1.14)
≥ 6 years			
No. of cases	112	112	112
Person-years	1006	1006	1006
Age-adjusted HRs	1.03 (1.01–1.06)	1.40 (1.12–1.76)	1.10 (1.03–1.18)
Multivariable adjusted HRs	1.03 (1.01–1.06)	1.32 (1.03–1.69)	1.07 (1.01–1.15)

Multivariable adjustments included diabetes duration, smoking status, eGFR, HDL, aspirin, and mean HbA1c

**Fig. 2:**
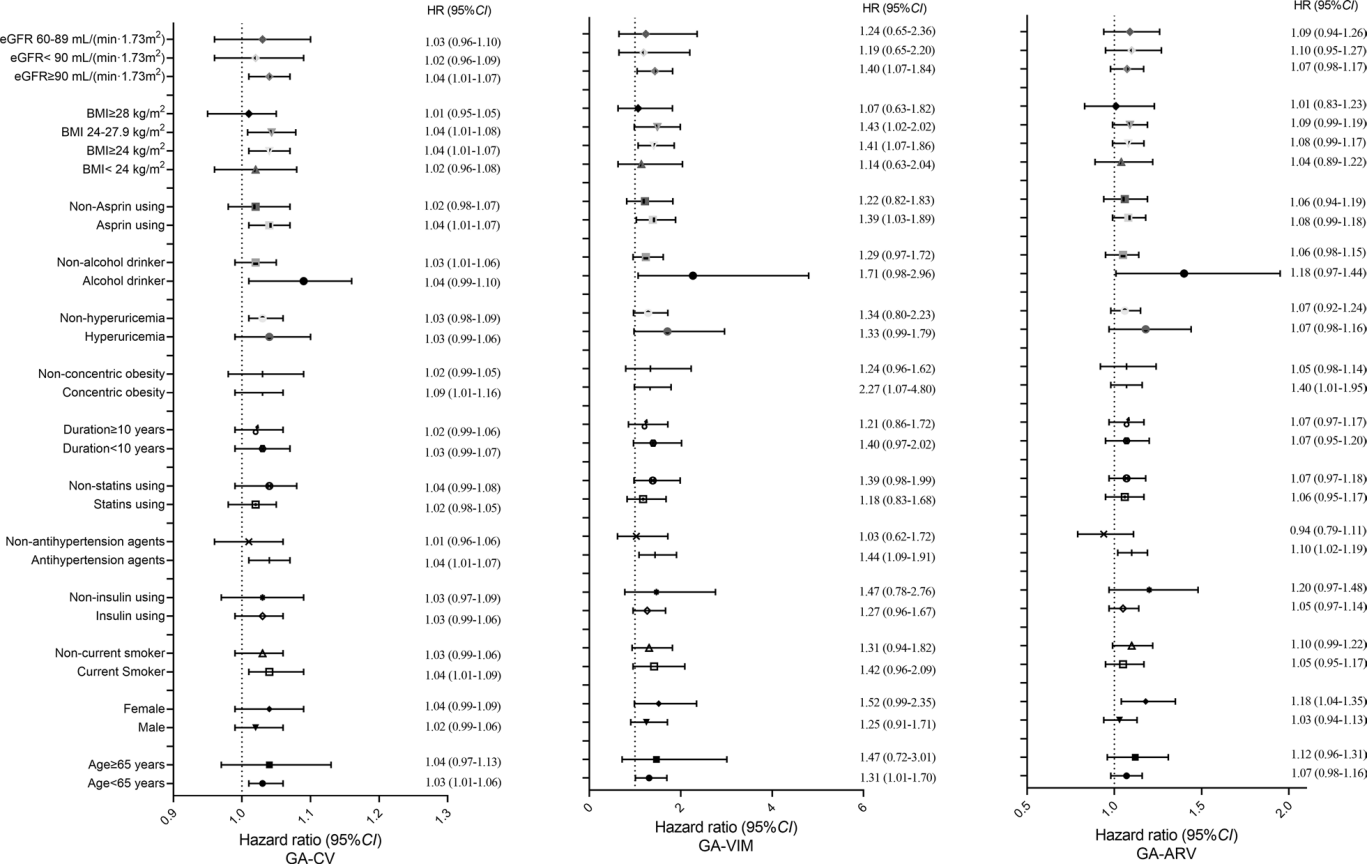
Hazard ratios for progression of LEAD risk base on different GA variability indicators in different subgroups

When stratified analyses were utilized, the positive association between GA-CV and the risk of the primary outcome was consistent among most but not all of subgroups (Fig. 2). Significant associations between GA-CV and the primary outcome were detected among patients with current smoking status, current alcohol drinking status, antihypertensive medication, use of aspirin and without hyperuricemia.

Associations between HbA1c-CV and the risks of the primary outcome are presented in Additional file 1: Table S1. There was no significant association between visit-to-visit variability of HbA1c and the risk of the primary outcome.

For the analysis of the secondary outcome (Additional file 1: Table S2), although the trends across tertiles of GA variability metrics in relation to incident LEAD were not significant (all *P* > 0.05), the linear association between GA-CV, GA-VIM and incident LEAD remained significant with multivariable adjustments (HR 1.04 (1.01–1.07) for CV and 1.44 (1.05–1.97) for VIM). Similar results were observed for the outcome of progressive LEAD with additionally GA-ARV significantly associated with the outcome as well.

## Discussion

In this prospective cohort study, we reported the positive association of long-term impact of GA-CV with the risk of LEAD among patients with type 2 diabetes, independent of HbA1c. We provided new insights into the clinical interpretation and evidence of GA. GA-CV may be a suitable metric for long-term glycemic variability, and it can be used as a supplementary measurement to conventional glycemic markers (such as mean glucose and HbA1c) during the comprehensive management of diabetic complications.

### Clinical implications of GA and GA variability in other studies

GA itself was reported to be closely related to cardiovascular disease and its subtypes in patients with diabetes [16] and even among subjects without diabetes or those with normal HbA1c levels [17], suggesting that GA has a potential advantage in predicting diabetic macrovascular complications. Moreover, GA may have other clinical implications other than diabetic complications such as a potential biomarker to predict the effects of clopidogrel antiplatelet therapy in ACS patients [18]. Limited evidence supported the association between visit-to-visit variability of GA and diabetic complications. June et al. [19] enrolled 498 patients with type 2 diabetes for more than 2 years of follow-up, and found that visit-to-visit variability of GA was significantly correlated with cardiac autonomic neuropathy. Another study involving 369 patients with type 2 diabetes by Park et al. [20] found that a higher visit-to-visit variability of GA was associated with a higher risk of diabetic nephropathy. Here, we demonstrated a significant association between GA variability and LEAD, which has broadened the clinical implications of GA. Meanwhile, survival analysis at different time points showed that GA variability may have a potential impact on LEAD within 4 years and over, indicating that this is an optimal long-term biomarker.

### Advantages of GA when compared to HbA1c

Compared with HbA1c, GA is not affected by hemoglobin, and can more accurately reflect the glycemic control in cases of anemia, hemoglobinopathy, dialysis [21], and pregnancy [22]. GA can reflect glycemic control in a relatively short period of time (2–3 weeks) when blood glucose deteriorates and improves alternately within a short period of time. HbA1c often remains unchanged while GA is reflective of this kind of glycemic fluctuation, suggesting that multiple measurements of GA are comparable to HbA1c with more information about long-term glycemic fluctuations. Koga et al. [7] regularly measured the GA and HbA1c values of patients with diabetes within one year and calculated the CV of these two metrics respectively. They found that GA-CV was significantly higher than HbA1c-CV, which was similar to our findings.

### Comparisons of different metrics of glycemic variability

We calculated several metrics in the present study including CV, VIM and ARV of GA and HbA1c based on at least 3 measurements within 2 years prior to baseline as indicators for evaluating long-term glycemic fluctuations. Notably, SD, CV, adj-SD, VIM, ARV and variability scores have all been used in previous studies [12, 23, 24]. Of these metrics, SD and adj-SD are affected by the average value and cannot fully reflect the glycemic variability; CV is not affected by the average value and is an ideal indicator for glycemic variability; VIM adjusts the effect of the average value on SD. There is a close association between ARV and variability scores, while variability score requires more times of measurement. Therefore, CV, VIM and ARV were selected as the exposures in the present study. Our findings supported GA-CV as a competitive marker in relation to LEAD, especially among type 2 diabetes patients with a moderate baseline level of HbA1c (mean level around 8%).

### Underlying mechanisms and potential bias of GA

The mechanism of an increased risk of macrovascular complications caused by increased long-term blood glucose fluctuations is not yet clear. Studies have potentially supported the hypothesis of oxidative stress [25], endothelial dysfunction [26] and the subsequent chronic inflammation caused by blood glucose variability. Basic scientists have shown that exposure to oscillating glucose was more deleterious than constant high glucose and induced a metabolic memory after glucose normalization [27]. This may partly explain why a patient with larger glycemic variability may be more likely to develop diabetic complications than one with constant hyperglycemia even when they have a same value of HbA1c. Although socioeconomic factors were not assessed in this study, we may assume a large discrepancy in these factors such as adherence to treatment, self-efficacy in diabetes management, and quality of life between patients with and without a stable glycemic control [28]. However, two studies have raised concerns about the potential impact of visceral adiposity on GA. Measurements of GA may be underestimated among obese patients, especially among those with visceral obesity [29, 30]. To address this concern, we have considered waist circumference as a potential confounding factor in the analysis. Waist circumference was not included in the model when we used a backward stepwise selection procedure. It seemed that visceral adiposity was not involved in the association between GA and LEAD in this study, but we expect more evidence supporting our findings.

### Strengths and limitations

The strength of this study included the originality, the prospective study design and the robust analysis. Limited studies have investigated the association between visit-to-visit variability of GA and the risk of LEAD in patients with type 2 diabetes. There are some limitations that need to be pointed out. First, this was a single center study with a relatively small sample size and few outcome cases. Second, our analyses adjusted for some confounding factors, but unmeasured factors such as other related chronic diseases, dietary factors, and physical activity could not be evaluated. Moreover, the index date of the primary outcome was determined by the date of lower extremity vascular ultrasonography. It is thus not yet possible to accurately obtain the time of lesion progression. However, the median follow-up time interval (Q1, Q3) in this study was 1.14 (0.85, 1.97) years, which is in line with the screening frequency recommended in guidelines for patients under a high risk of LEAD [31]. The subjects of this study were patients who had 3 or more HbA1c and GA measurements within 2 years, and had regular lower limb vascular ultrasonographic follow-ups. The compliance of this cluster of patients was higher than that of the general population with type 2 diabetes, which may account for a potential selection bias. Further retrospective real-world data with a large sample size are of great interest to validate our findings.

## Conclusions

In conclusion, visit-to-visit variability of GA predicts LEAD risk among patient with type 2 diabetes, independent of HbA1c levels. In contrast, visit-to-visit variability of HbA1c was not an independent factor for LEAD among patients with type 2 diabetes. Visit-to-visit variability of GA may be closely related to LEAD among patients with type 2 diabetes.

## Supplementary Information

**Additional file 1: Table S1. **Hazard ratios for the primary outcome based on different HbA1c variability metrics. **Table S2. **Hazard ratios for the secondary outcome based on different GA variability metrics. **Figure S1. **Flow chart of the study population.

## Data Availability

The datasets generated and/or analyzed in the current study are not publicly available but are available from the corresponding author on reasonable request.

## Abbreviations

ARV: Average real variability
BMI: Body mass index
CI: Confidence interval
CKD-EPI: Chronic Kidney Disease Epidemiology Collaboration equation
CV: Coefficient of variation
DPP-4: Dipeptidyl peptidase-4
eGFR: Estimated glomerular filtration rate
FCP: Fasting C peptide
FPG: Fasting blood glucose
GA: Glycated albumin
GLP-1: Glucagon-like peptide-1
HbA1c: Glycated hemoglobin A1c
HDL-C: High density lipoprotein cholesterol
HR: Hazard ratios
IMT: Intima-media thickness
LDL-C: Low-density lipoprotein cholesterol
LEAD: Lower extremity atherosclerotic disease
RAAS: Renin–Angiotensin–Aldosterone System
SBP: Systolic blood pressure
Scr: Serum creatinine
SD: Standard deviation
SGLT-2: Sodium-dependent glucose transporters-2
TC: Total cholesterol
TG: Triglycerides
UA: Uric acid
VIM: Variability independent of the mean
