# Supplementary material for: Visit-to-visit variability of glycated albumin was associated with incidence or progression of lower extremity atherosclerotic disease
Source: Cardiovasc Diabetol. 2020 Dec 10;19:211. doi: 10.1186/s12933-020-01187-1 (PMC7731472; doi:10.1186/s12933-020-01187-1)
Supplement: Supplementary file 1 — Additional file 1: Table S1. Hazard ratios for the primary outcome based on different HbA1c variability metrics. Table S2. Hazard ratios for the secondary outcome based on different GA variability metrics. Figure S1. Flow chart of the study population. [file 12933_2020_1187_MOESM1_ESM.docx]

**Additional Appendix**

**Additional Tables**

**Table S1.** Hazard ratios for the primary outcome based on different HbA1c variability metrics

**Table S2.** Hazard ratios for the secondary outcome based on different GA variability metrics

**Additional Figures**

**Figure S1.** Flow chart of the study population

**Table S1.** Hazard ratios for progression of LEAD based on different HbA1c variability metrics

|  | HbA_1c_ variability indicators tertiles | | | *P* value for trend | Per 1-unit increase |
| --- | --- | --- | --- | --- | --- |
|  | T1 | T2 | T3 |  |  |
| **CV** | ＜4.69 | 4.69-7.71 | ≥7.72 | - | - |
| No. of participants | 146 | 144 | 146 | - | - |
| No. of cases | 29 | 36 | 47 | - | - |
| Person-years | 533 | 484 | 475 | - | - |
| Age-adjusted HRs | 1.00 | 1.39(0.85-2.27) | 1.90(1.19-3.03) | 0.024 | 1.03(1.01-1.06) |
| Multivariable-adjusted HRs | 1.00 | 1.48(0.90-2.43) | 1.92(1.20-3.07) | 0.025 | 1.03(1.01-1.06) |
| **VIM** | ＜0.14 | 0.14-0.22 | ≥0.23 | - | - |
| No. of participants | 146 | 144 | 146 | - | - |
| No. of cases | 29 | 37 | 46 | - | - |
| Person-years | 531 | 488 | 473 | - | - |
| Age-adjusted HRs | 1.00 | 1.40(0.86-2.28) | 1.85(1.16-2.94) | 0.036 | 2.72(1.12-6.57) |
| Multivariable-adjusted HRs | 1.00 | 1.37(0.84-2.25) | 1.81(1.13-2.90) | 0.047 | 2.39(0.99-5.73) |
| **ARV** | ＜0.40 | 0.40-0.82 | ≥0.83 | - | - |
| No. of participants | 148 | 143 | 145 | - | - |
| No. of cases | 30 | 33 | 50 | - | - |
| Person-years | 523 | 494 | 475 | - | - |
| Age-adjusted HRs | 1.00 | 1.16(0.71-1.90) | 1.82(1.15-2.94) | 0.021 | 1.28(1.01-1.61) |
| Multivariable-adjusted HRs | 1.00 | 1.10(0.67-1.81) | 1.75(1.10-2.78) | 0.029 | 1.22(0.96-1.54) |

Adjustments included diabetes duration, smoking status, eGFR, HDL, and aspirin.

**Table S2.** Hazard ratios for secondary outcome based on different GA variability metrics

|  | GA variability indicators tertiles | | | *P* value for trend | Per 1-unit increase |
| --- | --- | --- | --- | --- | --- |
|  | T1 | T2 | T3 |  |  |
| **Incident LEAD** |  |  |  |  |  |
| **CV** | <6.24 | 6.24-11.4 | ≥11.5 | - | - |
| No. of participants | 65 | 65 | 64 | - | - |
| No. of cases | 22 | 21 | 32 | - | - |
| Person-years | 239 | 249 | 205 | - | - |
| Age-adjusted HRs | 1.00 | 1.04(0.57-1.92) | 1.89(1.09-3.30) | 0.034 | 1.04 (1.01-1.06) |
| Multivariable-adjusted HRs | 1.00 | 0.93(0.50-1.72) | 1.51(0.85-2.69) | 0.180 | 1.04 (1.01-1.07) |
| **VIM** | <0.52 | 0.52-0.99 | ≥1.00 | - | - |
| No. of participants | 64 | 66 | 64 | - | - |
| No. of cases | 20 | 21 | 34 | - | - |
| Person-years | 224 | 261 | 206 | - | - |
| Age-adjusted HRs | 1.00 | 0.95(0.51-1.77) | 2.06(1.17-3.65) | 0.007 | 1.46 (1.11-1.92) |
| Multivariable-adjusted HRs | 1.00 | 0.96(0.50-1.82) | 1.62(0.87-3.04) | 0.138 | 1.44 (1.05-1.97) |
| **ARV** | <1.45 | 1.45-3.17 | ≥3.18 | - | - |
| No. of participants | 64 | 64 | 66 | - | - |
| No. of cases | 21 | 21 | 33 | - | - |
| Person-years | 228 | 249 | 215 | - | - |
| Age-adjusted HRs | 1.00 | 1.10(0.59-2.04) | 1.89(1.08-3.32) | 0.044 | 1.13 (1.02-1.24) |
| Multivariable-adjusted HRs | 1.00 | 0.98(0.51-1.86) | 1.50(0.80-2.82) | 0.281 | 1.10 (0.98-1.24) |
| **Progressive LEAD** |  |  |  |  |  |
| **CV** | <8.18 | 8.15-13.4 | ≥13.5 | - | - |
| No. of participants | 80 | 81 | 81 | - | - |
| No. of cases | 6 | 14 | 17 | - | - |
| Person-years | 288 | 248 | 270 | - | - |
| Age-adjusted HRs | 1.00 | 2.79(1.07-7.30) | 3.09(1.21-7.88) | 0.052 | 1.05 (1.01-1.10) |
| Multivariable-adjusted HRs | 1.00 | 2.85(1.04-7.78) | 3.01(1.13-7.97) | 0.065 | 1.05 (1.01-1.10) |
| **VIM** | <0.69 | 0.69-1.17 | ≥1.18 | - | - |
| No. of participants | 81 | 81 | 80 | - | - |
| No. of cases | 6 | 16 | 15 | - | - |
| Person-years | 294 | 266 | 246 | - | - |
| Age-adjusted HRs | 1.00 | 3.40(1.31-8.80) | 3.18(1.21-8.35) | 0.031 | 1.65(1.14-2.40) |
| Multivariable-adjusted HRs | 1.00 | 3.07(1.15-8.22) | 2.85(1.01-8.08) | 0.070 | 1.55 (1.02-2.36) |
| **ARV** | <2.25 | 2.25-3.92 | ≥3.93 | - | - |
| No. of participants | 81 | 81 | 80 | - | - |
| No. of cases | 8 | 10 | 19 | - | - |
| Person-years | 295 | 164 | 247 | - | - |
| Age-adjusted HRs | 1.00 | 1.48(0.58-3.78) | 2.85(1.23-6.59) | 0.033 | 1.16 (1.06-1.27) |
| Multivariable-adjusted HRs | 1.00 | 1.37(0.53-3.58) | 2.57(0.99-6.61) | 0.108 | 1.14 (1.02-1.26) |

Multivariable adjustments included diabetes duration, smoking status, eGFR, HDL, aspirin, and mean HbA1c.


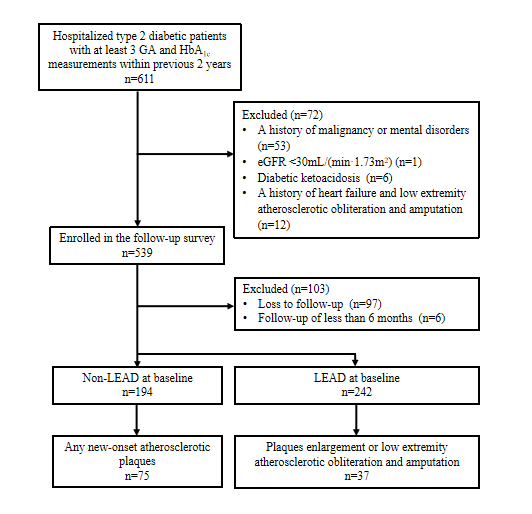


**Figure S1.** Flow chart of the study population
